# Combined association between physical activity and sedentary behavior on the cardiometabolic risk score in students

**DOI:** 10.1590/1984-0462/2026/44/2025033

**Published:** 2026-01-19

**Authors:** Lidyane Ferreira Zambrin, Julio Cesar da Costa, Vinícius Muller Reis Weber, Maria Raquel de Oliveira Bueno, Marcelo Romanzini, Enio Ricardo Vaz Ronque

**Affiliations:** a Universidade Federal do Mato Grosso do Sul, Corumbá, MS, Brazil. Universidade Federal do Mato Grosso do Sul Corumbá MS Brazil; b Universidade Estadual de Londrina, Londrina, PR, Brazil. Universidade Estadual de Londrina Londrina PR Brazil

**Keywords:** Motor activity, Healthy lifestyle, Adolescent, Cardiometabolic risk factors, Atividade motora, Estilo de vida saudável, Adolescente, Fatores de risco cardiometabólico

## Abstract

**Objective:**

This study aimed to verify the combined association of moderate-to-vigorous physical activity (MVPA) with sedentary behavior (SB) on the cardiometabolic risk factors (CMR) of adolescents.

**Methods:**

A cross-sectional study was performed in Londrina/Paraná/Brazil between October 2015 and May 2017 with a representative sample of adolescents of both sexes from the sixth year of primary public schools. The participants consisted of 367 adolescents, with a mean age of 11.8±0.6 years. For the cardiometabolic risk score, the following measures were used: waist circumference (WC), systolic and diastolic blood pressure, and cardiorespiratory fitness (CRF). MVPA and SB were measured through accelerometry. Multiple linear regression controlled for chronological age was used, adopting p<0.05.

**Results:**

For boys, the “High MVPA/Low SB” group compared to the “Low MVPA/High SB” group had significantly lower WC and CMR z-score. For CRF, the “High MVPA/Low SB” group presented better values when compared to “Low MVPA/Low SB” and “Low MVPA/High SB” groups. Adjusted analyses by chronological age show positive association between “High MVPA/Low SB” and “Low MVPA/High SB”, demonstrating that the “High MVPA/Low SB” group has lower WC and lower CMR. Negative association in CRF was also observed between “High MVPA/Low SB” and both “Low MVPA” groups, regardless of SB.

**Conclusions:**

Being physically active is associated with lower cardiometabolic risk score regardless of SB, especially in boys. The current study suggests that adolescents should be encouraged to increase physical activity and reach MVPA recommendations.

## RESUMO

**Objetivo:**

Este estudo teve como objetivo verificar a associação combinada entre atividade física de intensidade moderada a vigorosa (AFMV) e comportamento sedentário (CS) nos fatores de risco cardiometabólico (CMR) de adolescentes.

**Métodos:**

Foi realizado um estudo transversal em Londrina/Paraná/Brasil entre outubro de 2015 e maio de 2017 com uma amostra representativa de adolescentes de ambos os sexos, do sexto ano do ensino fundamental de escolas públicas. Os participantes foram 367 adolescentes, com idade média de 11,8±0,6 anos. Para o escore de risco cardiometabólico foram utilizadas as seguintes medidas: circunferência da cintura (CC), pressão arterial sistólica e diastólica e aptidão cardiorrespiratória (ACR). AFMV e CS foram mensurados por acelerometria. Foi utilizada Regressão linear múltipla controlada por idade cronológica, adotando-se p<0,05.

**Resultados:**

Para os meninos, o grupo “AFMV alta/CS baixo” em comparação ao grupo “AFMV baixa/CS alto” apresentou escore Z de CC e CMR significativamente menor. Para a ACR, o grupo “AFMV alta/CS baixa” apresentou melhores valores quando comparado aos grupos “AFMV baixa/CS baixo” e “AFMV baixa/CS alto”. Análises ajustadas por idade cronológica mostram associação positiva entre “AFMV alta/CS baixo” e “AFMV baixa/CS alto”, demonstrando que o grupo “AFMV alta/CS baixa” tem menor CC e menor CMR. Associação negativa na ACR foi observada entre os grupos “AFMV alta/CS baixo” e ambos os grupos de “AFMV baixa”, independentemente do CS.

**Conclusões:**

Ser fisicamente ativo está associado a menor escore de risco cardiometabólico, independentemente do CS, especialmente em meninos. Sugere-se que os adolescentes pratiquem mais atividade física e atinjam as recomendações de AFMV.

## INTRODUCTION

The regular practice of physical activity (PA) provides numerous health benefits. In recent years, PA and sedentary behavior (SB) and their independent associations with negative health outcomes have become an area of increasing concern, especially in children and adolescents.^1^

Although some studies show that moderate-to-vigorous physical activity (MVPA) may have a lower cardiometabolic risk in children and adolescents than excessive SB time, further research is needed to verify the association of these combined behaviors with cardiometabolic risk.^2,3^ Cross-sectional studies have reported that MVPA is independently associated with adiposity indicators in children. However, time spent in SB is not associated with adiposity after adjusting for MVPA, showing the possible impact of MVPA on the association of SB and health related factors.^2,4^ Likewise, no association was found between total SB and pattern of SB with cardiometabolic risk factors in children and adolescents. On the other hand, the time watching television (TV) and low MVPA were independently associated with cardiometabolic risk factors.^5^

Finally, Ekelund et al. analyzed children and adolescents and showed that long time in MVPA was associated with better cardiometabolic profile regardless of time spent in SB.^6^ In this sense, some studies started to investigate the combined association between physical activity and SB with health risk factors in order to understand the lifestyle of children and adolescents and its association with health.^7,8,9,10^

However, most studies that evaluated the combined association of MVPA with SB concentrated only on isolated health risk factors (i.e. obesity, fitness) in pediatric populations.^7,8,9,10^ The use of a more comprehensive indicator, grouping cardiometabolic risk factors, seems to be a better cardiovascular risk marker in young people, when compared to isolated risk factors, probably due to the daily changes in risk factors and PA.^11^

In children and adolescents, cardiometabolic risk refers to a set of metabolic conditions such as abdominal adiposity, altered blood glucose, high blood pressure and dyslipidemia, which can be analyzed separately or using a metabolic score.^12,13^ In this sense, cardiometabolic risk factors in children and adolescents have been frequently studied in literature; however, the combination of these risk factors has been investigated only more recently. Additionally, studies using subjective methods, for example questionnaires, to analyze the combined association of PA and SB, have found results different from those using objective measures, such as accelerometers. With the use of accelerometry, MVPA seems to present a protective factor against CMR, regardless of time spent in SB, a result that has not been demonstrated by studies using self-reported data.^10,14,15,16^

So, a combined classification between PA and SB would be essential to establish future public health strategies, aiming to increase PA in childhood to reduce the prevalence of coronary and metabolic diseases in adulthood.^17^ Thus, the aim of this study was to verify the combined association between MVPA and SB on the isolated and grouped cardiometabolic risk factors of adolescents.

## METHOD

This is a school-based cross-sectional study, involving adolescents (both sexes) aged 10–14 years enrolled in the sixth grade of public elementary schools of Londrina, Paraná, Brazil. The city of Londrina has a population of 555,965 people, according to the last census, in 2022, and the Municipal Human Development Index is 0.778 (considered high development) and a Gross Domestic Product of R$ 40,636.89.

**Figure 1.**
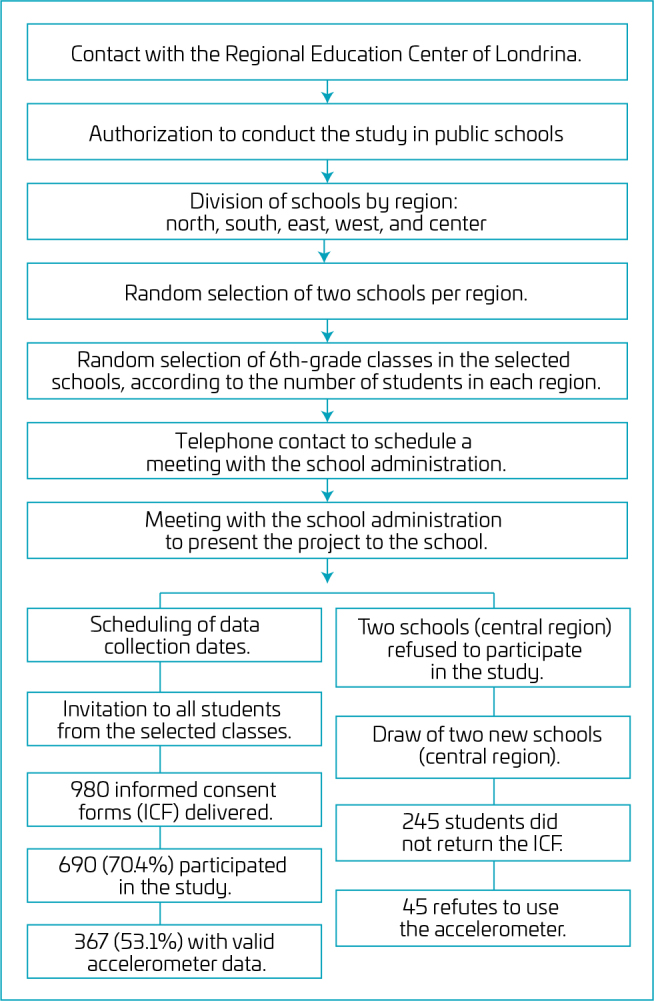
Flowchart of the sample selection process.

The sixth year in the Brazilian school system corresponds to the year in which the adolescent completes 11 years, but 11% repeated one year or more. Regarding the sampling process, all the public schools of the city were divided into five regions (north, south, east, west, and center), and two schools were randomly selected from each region. Thus, classes were randomly selected from the selected schools, and all the students of the selected classes were invited to participate in the study. The sample selection process can be seen in Figure 1. Both the students and their guardians signed the informed consent to participate. Exclusion criteria were students who had any physical limitations, those being treated for any disease or injury during the study, those who refused to use the accelerometer, or those who did not have authorization signed by their parents or guardians. The previous sample size calculation showed that n=175 is adequate to attend the follow statistical power: f2=0,15; 1-β=0,99; α=0,05; total number of predictors=8 (GPower 3.1.9.4). The study was approved by the Human Research Ethics Committee of our institution according to decision No. 1.281.324.

In total, 980 adolescents were invited to participate in the study. Refusals and losses totaled 290 individuals (29.5%), of which 245 were excluded for not having delivered the informed consent form signed by their parents or guardians and 45 for not agreeing to use the accelerometer. Of the total of 690 adolescents followed by accelerometers, 367 presented valid data (52.1% girls), and were included in the analyses (Table 1).

**Table 1.** Descriptive characteristics of participants. Values expressed as mean and standard deviation.

	All (367)	Boys (176)	Girls (191)	t	p-value
Chronological age (years)	11.8±0.6	11.8±0.7	11.7±0.6	-1283	0.200
Peak Height Velocity (years)	-2.0±0.7	-1.8±0.8	-2.2±0.5	-5066	<0.001^*^
Body mass (kg)	46.1±11.8	45.6±12.2	46.6±11.5	0.862	0.389
Height (cm)	151.6±8.0	150.6±8.5	152.4±7.4	2180	0.030^*^
BMI (kg/m^2^)	19.9±4.2	19.9±4.3	19.9±4.1	0.105	0.917
WC (cm)	67.4±9.1	68.7±9.4	66.2±8.7	-2715	0.007^*^
SBP (mmHg)	106.7±10.1	106.0±9.1	107.3±10.8	1216	0.225
DBP (mmHg)	63.6±8.1	63.0±8.3	64.1±7.9	1248	0.213
MAP (mmHg)	85.1±8.4	84.5±8.0	85.7±8.7	1334	0.183
CRF (km/h)	9.7±0.9	10.0±0.9	9.4±0.7	-6938	<0.001^*^
MVPA (min/week)	279.8±154.6	328.9±178.1	234.5±111.9	-6128	<0.001^*^
SB (min/week)	4111.7±1795.9	4161.3±1877.1	4066.1±1721.3	-0.507	0.613
Normal weight (%)	65.6	61.4	69.4	2654	0.103
Overweight (%)	34.4	38.6	30.6		

BMI: body mass index; WC: waist circumference; SBP: systolic blood pressure; DBP: diastolic blood pressure; MAP: mean blood pressure; CRF: cardiorespiratory fitness; MVPA: moderate/vigorous physical activity; SB: sedentary behavior.

*p<0.05.

Body mass, height measurements were collected using standardized procedures with portable digital scale (Seca, Hamburg, Germany) and portable stadiometer with 0.1 cm precision (Harpenden Holtain Ltd, Crymch, Dyfed, United Kingdom).^18^ From this information, body mass index (BMI) was determined by the body mass/height^2^ quotient (kg/m^2^). Waist circumference (WC) was measured using non-elastic anthropometric tape with accuracy of 0.1 cm according to recommended procedures.^19^

Nutritional status was assessed using the cut-off points suggested by the International Obesity Task Force, with students classified as normal weight and overweight.^20^

Biological maturation was estimated by assessing somatic maturation, by determining the distance in years that the individual was at the peak height velocity (PHV), based on mathematical models using anthropometric measures (height, sitting height, length of the lower limbs and body mass), age and sex, as described below.^21^

Systolic blood pressure (SBP) and diastolic blood pressure (DBP) were obtained using OMRON digital device, model HEM-742, with cuffs appropriate for each subject’s arm circumference. This equipment has been validated for adolescents by Christofaro et al.^22^ Prior to measurements, subjects were instructed to empty their bladder, and to remain seated in a chair, at rest, for 5 min. Three BP measurements were carried out with an interval of two minutes between them, with the cuff placed on the right arm. The subject remained in the sitting position, legs uncrossed, feet flat on the floor, and back against the chair. The arm was required to be at the height of the heart (level of the midpoint of the sternum or 4^th^ intercostal space), supported, with the palm of the hand facing upwards. The mean value of the last two SBP and DBP measures were considered for the analysis.

To estimate cardiorespiratory fitness (CRF), the 20-meter Shuttle Run (SR-20m) test was used, carried out in a sports court of schools, in a space with a distance of 20 meters demarked between two lines. Students were required to move continuously from one end to the other, progressively, until exhaustion, guided by a sound recording. The initial running speed was 8.5 km/h with increments of 0.5 km/h at each one-minute stage. The performance of the test, as well as the criterion adopted for its completion, were in accordance with recommendations of Léger et al., and the speed in the final stage was adopted for the analyses.^23^

Variables reflecting abdominal adiposity (WC), systolic blood pressure (SBP), diastolic blood pressure (DBP), and cardiorespiratory fitness (SR-20m) were combined to calculate a cardiometabolic risk score. These variables were standardized in z-score (Z=(X–μ)/σ, where X is the individual mean, μ is the population mean, and σ is the population standard deviation) units according to sex and age. Finally, a combined cardiomet-abolic risk factor was calculated from the sum of the z-scores of variables mentioned above. The sign of the CRF variable was inverted for this calculation (CRF)^1^ since it presents an opposite direction with respect to risk compared to the other variables.^13^

Accelerometry measurements were obtained using ActiGraph monitors (ActiGraph, Pensacola, FL, USA), models GT3X and GT3X-Plus. Participants were instructed to use the accelerometer for seven consecutive days during the period in which they were awake, with instructions for removing equipment only during bathing, water activities, and sleeping. Accelerometers were programmed to collect data in epochs of 1 second (GT3X) and 30 Hz (GT3X+), which were later reintegrated in epochs of 15 seconds.

After the monitoring period, the equipment was collected, and data were stored in the ActiLife software (version 6.8.2). Individuals who obtained at least four valid days of data recorded by the accelerometer were included in the analyses, that is, at least eight hours of use per day (≥ 480 minutes/day), including at least one valid weekend day. The non-use criterion was based on 60 minutes of consecutive zeros.

The cut-off points of Evenson et al. were used to classify the counts recorded by accelerometers into minutes of sedentary activity (0–25 counts.15 sec^-1^) and MVPA (≥574 counts.15sec^-1^).^24^ For the combined analyses, the sample was standardized in z-score units by sex, and adolescents with positive values were classified as high MVPA/SB and those with negative values as low MVPA/SB, thus forming four groups of analysis: (1) High MVPA/Low SB; (2) High MVPA/High SB; (3) Low MVPA/Low SB; (4) Low MVPA/High SB.

Data normality was verified through asymmetry and kurtosis. Data are described by sex using mean values, standard deviation, and 95% confidence interval and frequency distribution. Student’s *t-*test for independent samples and chi-square were used to detect differences between boys and girls. For comparisons between combined MVPA and SB groups with CMR by sex, analysis of covariance (ANCOVA) was used, controlled for chronological age, followed by Bonferroni’s post hoc to identify differences. To analyze the combined association of MVPA and SB with cardiometabolic risk factors by sex, Multiple Linear Regression controlled for chronological age was used, and, for this purpose, *dummy* variables were created, and the reference category was the “High MVPA and Low SB” group. Analyses were individually performed between groups and reference categories. All analyses were performed using the Statistical Package for the Social Sciences — SPSS 25.0 (IBM SPSS Statistics for Windows, 25.0, IBM Corp., Armonk, NY), adopting p<0.05.

## RESULTS

Table 1 presents the description of anthropometric and cardiometabolic risk of participants. Variables height, WC, CRF and MVPA demonstrated statistically significant differences between boys and girls, in which girls presented greater height (152.4±7.4 *vs.* 150.6±8.5), and lower WC (66.2±8.7 *vs*. 68.7±9.4), CRF (9.4±0.7 *vs*. 10.0±0.9), and MVPA (234.5±111.9 *vs*. 328.9±178.1).

In the ANCOVA analyses (Table 2), boys categorized as “High MVPA/Low SB” and “High MVPA/High SB” presented lower WC (F=5.606; p=0.001) and CMR (F=5.596; p=0.001) value when compared to these in the “Low MVPA/High SB” group. Both combined “High MVPA” groups showed 5% (10.2 *vs*. 9.7; p<0.001) to 8% (10.3 *vs*. 9.5; p<0.001) higher CRF when compared to “Low MVPA” groups, regardless of SB in boys.

**Table 2.** Comparison of cardiometabolic risk factors between moderate/vigorous physical activity and sedentary behavior by sex. Values expressed as estimative mean (95%CI).

	High MVPA/Low SB	High MVPA/High SB	Low MVPA/Low SB	Low MVPA/High SB	F	p-value
Boys	(n=47)	(n=47)	(n=60)	(n=22)		
WC (cm)	66.7 (64.0–69.5)^*^	66.2 (63.9–68.4)	70.1 (67.7–72.6)	74.6 (70.2–79.1)	4.302	**0.006^‡^**
SBP (mmHg)	106.8 (104.0–109.7)	103.7 (101.2–106.2)	106.3 (103.8–108.7)	108.8 (105.4–112.1)	1.148	0.331
DBP (mmHg)	63.1 (60.4–65.9)	62.3 (59.7–64.8)	62.7 (60.7–64.8)	65.2 (62.7–67.8)	0.296	0.828
CRF (km/h)	10.3 (10.1–10.6)^†^	10.2 (10.0–10.5)^†^	9.7 (9.5–10.0)	9.5 (9.1–9.8)	7.848	**<0.001^‡^**
CMR (z-score)	-0.5 (-1.3–0.4)	-0.9 (-1.7–0.1)^*^	0.4 (-0.3–1.1)	1.7 (0.7–2.8)	3.888	**0.010^‡^**
**Girls**	**(n=25)**	**(n=27)**	**(n=99)**	**(n=40)**		
WC (cm)	66.7 (63.2–70.3)	66.3 (62.6–70.1)	66.4 (64.6–68.2)	65.0 (62.6–67.4)	0.246	0.854
SBP (mmHg)	106.2 (100.6–111.8)	109.3 (104.6–114.0)	107.1 (105.0–109.1)	107.3 (104.0–110.6)	0.174	0.914
DBP (mmHg)	64.2 (60.4–67.9)	65.2 (61.9–68.6)	63.9 (62.4–65.5)	63.6 (61.4–65.9)	0.350	0.789
CRF (km/h)	9.4 (9.2–9.7)	9.5 (9.2–9.9)	9.3 (9.2–9.5)	9.4 (9.1–9.6)	0.797	0.497
CMR (z-score)	-0.1 (-1.3–1.1)	0.1 (-1.1–1.3)	0.0 (-0.5–0.6)	-0.2 (-1.0–0.7)	0.013	0.998

Models adjusted by chronological age and peak height velocity.

MVPA: moderate/vigorous physical activity; SB: sedentary behavior; WC: waist circumference; SBP: systolic blood pressure; DBP: diastolic blood pressure; CRF: cardiorespiratory fitness; CMR: cardiometabolic risk score.

^*^difference to low moderate/vigorous physical activity and high sedentary behavior;

^†^difference to low moderate/vigorous physical activity and low sedentary behavior;

^‡^p<0.05.

**Figure 2.**
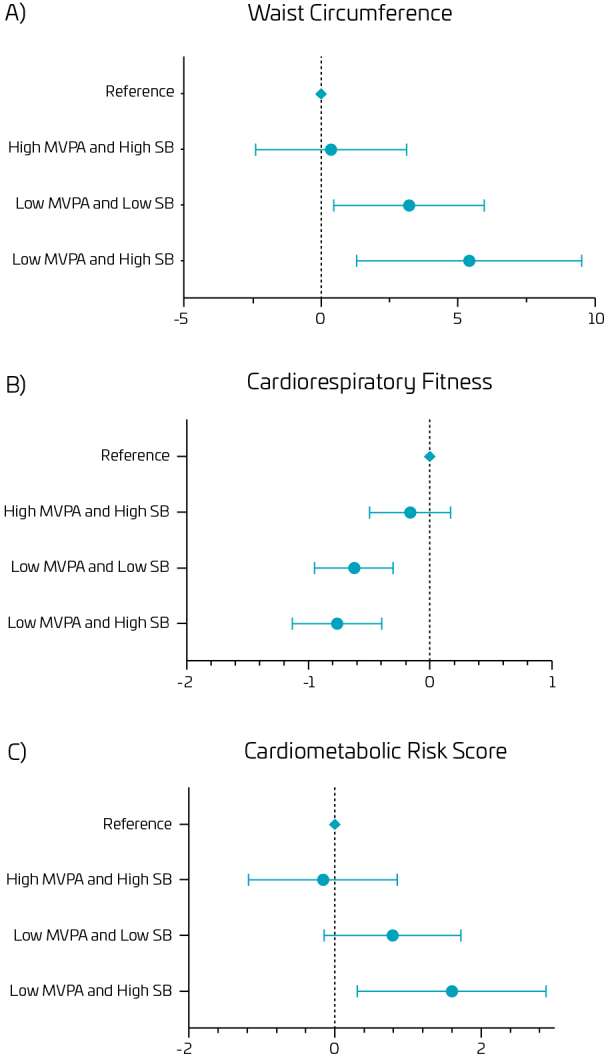
Combined relationship between moderate/vigorous physical activity and sedentary behavior with cardiometabolic risk factors in boys. Values presented as beta and coefficient interval (95%CI).

MVPA: moderate/vigorous physical activity; SB: sedentary behavior. High moderate/vigorous physical activity and low sedentary behavior; models adjusted by chronological age and peak height velocity.

Figure 2 presents information regarding the association between MVPA and SB groups with cardiometabolic risk factors in boys, adjusted by chronological age. Positive associations were observed between “High MVPA/Low SB” and “Low MVPA/High SB” groups, demonstrating that the “High MVPA/Low SB” group has 7.63 less WC z-score units and 2.25 less CMR z-score units, and negative associations between “High MVPA/Low SB” with “Low MVPA/Low SB” (β = -0.617) and “Low MVPA/High SB” groups (β=-0.880) for CRF.

## DISCUSSION

The main findings of the study showed that boys in the “High MVPA/Low SB” group and those in the “High MVPA/High SB” group had lower WC and CRF values when compared to participants in the “Low MVPA/High SB” group, demonstrating that, regardless of time spent in SB, the practice of PA becomes an important protective factor for isolated cardiometabolic risk factors, especially those related to body adiposity and physical fitness. Likewise, Marques et al. suggest that adolescents categorized as active/low sedentary were more likely of having higher overall physical fitness score than those categorized as inactive/high sedentary. Therefore, physically active children and adolescents tend to present benefits in several health parameters, including CRF.^25^

In this sense, previous studies using questionnaires and accelerometers to measure SB found results similar to those found in the present study.^3,10,26^ In contrast, Moore et al. found that both vigorous physical activity and SB were independently associated with CRF.^27^ In addition, Ekelund et al. and Santos et al. found inverse association between objectively measured SB and CRF.^28,29^ Furthermore, Cristi-Montero et al. reported that CRF appears to be more sensitive than other health parameters to detect any beneficial physiological alteration.^3^

In addition, our results demonstrate that only boys in the “High MVPA/Low SB” group presented 7.63 less WC z-score units and 2.25 less CMR z-score units when compared to the “Low MVPA/High SB” group, adjusted by chronological age and PHV. Previous studies have also found that high MVPA level was associated with reduced isolated cardiometabolic risk in children and adolescents,^2,6^ although the analyses were stratified by sex. The reduced number of girls who had z-score values above the average MVPA (High MVPA/Low SB and High MVPA/High SB) and the fact that they are less active than boys may be the reason for non-significant results in all variables analyzed.

These results contrast with some previous observations in adults, which suggest that objectively measured sedentary time is associated with harmful metabolic outcomes regardless of MVPA.^30^ Discrepancies in results between children and adults are still a matter of debate, but can be explained by many factors, including the investigated cardiometabolic results (children have healthier cardiometabolic risk profile than adults, reducing the variability between subjects) and due to the fact that total physical activity (cpm or steps.day^-1^) is generally higher in children than in adults. In addition, PA is highly variable in children,^6^ indicating that the combined associations of MVPA and SB with metabolic risk indicators (WC, BP, and CRF) should be further explored, especially through accelerometry.

Current PA and SB guidelines for young individuals recommend that children and adolescents accumulate at least 60 minutes of daily MVPA and limit leisure screen time to no more than 2 hours per day.^1,17^ Thus, the combined PA and SB results indicate that although reducing time spent in SB is important for health-related aspects, increasing MVPA practice seems to have a greater influence on reducing cardiometabolic risk in the boys in this study.

The strengths of this study stem from the large representative sample of elementary school students of Londrina; additionally, this is the first study to use objective MVPA and SB measurement and cardiometabolic risk score in a sample of Brazilian adolescents. The main limitation of the current study is its cross-sectional design, which makes it difficult to infer causality from these associations, and the fact that many students lack valid accelerometer data, despite showing no differences in descriptive characteristics (Supplementary Table). Additionally, it is important to highlight that the study was limited to investigating adolescents in a restricted age range of public schools in Londrina-Paraná, which presupposes some caution regarding the generalization of the results.

Finally, future studies should use longitudinal designs to observe the causality between the combination between PA and SB and cardiometabolic risk, in addition to considering the possible effects of biological maturation on the variables involved.

As practical applications, the present study demonstrates the importance of increasing the time spent in PA, specially of moderate and vigorous intensity, in order to decrease CMR. In addition, we highlight the importance of improving the environments where children and adolescents spend most of their time, such as schools and parks, as well as the adequate presence of structures for active travel, such as cycle paths and sidewalks, which can contribute to the promotion of MVPA in this population. This information could support intervention actions with the objective of increasing PA and improving CRF in young people, thus, minimizing public spending and providing better quality of life for this population. It could also contribute to the dissemination of knowledge to health professionals and parents or guardians regarding the importance of the time spent in PA and SB, particularly during adolescence.

Our results indicate that being physically active is associated with lower cardiometabolic risk score regardless of SB, especially in boys. In other words, high or low time spent in SB does not compensate for the cardiometabolic risk score when combined with low PA time (i.e., the “Low MVPA/High SB” and “Low MVPA/Low SB” groups presented higher risk for abdominal adiposity, cardiometabolic risk score and low cardiorespiratory fitness). On the other hand, the High MVPA group, regardless of time spent in SB, does not present greater risk (i.e., similar odds ratio in the “High MVPA/Low SB” and “High MVPA/High SB” groups). This suggests that higher PA levels may be more critical than lower SB levels for lower prevalence of abdominal adiposity, low cardiorespiratory fitness and cardiometabolic risk score, especially in young males. The findings of the present study suggest that adolescents should be encouraged to increase physical activity to reduce cardiometabolic risk.

## Data Availability

The database that originated the article is available with the corresponding author.
