# Supplementary material for: Combined association between physical activity and sedentary behavior on the cardiometabolic risk score in students
Source: Rev Paul Pediatr. 2026 Jan 19;44:e2025033. doi: 10.1590/1984-0462/2026/44/2025033 (PMC12815412; doi:10.1590/1984-0462/2026/44/2025033)
Supplement: Supplementary file 1 [file 1984-0462-rpp-44-e2025033-Supplementary-Table.docx]

**Supplementary Table –** Comparison of anthropometric, blood pressure, and cardiorespiratory variables between participants with valid and invalid accelerometer data by sex.

|  | **Boys** | | | | **Girls** | | | |
| --- | --- | --- | --- | --- | --- | --- | --- | --- |
|  | **Valid**  **n=176** | **Invalid**  **n=151** | **t** | **p** | **Valid**  **n=191** | **Invalid**  **n=172** | **t** | **p** |
| Chronological age (years) | 11.8 ±0.7 | 12.0 ±0.8 | 1.379 | 0.169 | 11.7 ±0.6 | 11.9 ±0.7 | 1.648 | 0.100 |
| Peak Height Velocity (years) | -1.8 ±0.8 | -1.7 ±0.8 | 1.233 | 0.218 | -0.0 ±0.7 | 0.0 ±0.7 | 0.997 | 0.320 |
| Body Mass (kg) | 45.7 ±12.2 | 47.8 ±14.8 | 1.345 | 0.180 | 47.3 ±12.6 | 47.6 ±12.3 | 0.239 | 0.811 |
| Height (cm) | 150.8 ±8.7 | 151.5 ±9.0 | 0.795 | 0.427 | 152.6 ±7.5 | 152.9 ±7.5 | 0.296 | 0.768 |
| BMI (kg/m^2^) | 19.9 ±4.2 | 20.4 ±4.8 | 1.078 | 0.282 | 20.1 ±4.3 | 20.2 ±4.5 | 0.219 | 0.827 |
| WC (cm) | 68.7 ±9.3 | 69.8 ±11.4 | 0.812 | 0.417 | 66.6 ±9.2 | 66.6 ±9.2 | 0.050 | 0.960 |
| SBP (mmHg) | 106.6 ±8.8 | 108.1 ±10.5 | 1.434 | 0.159 | 108.1 ±10.8 | 107.9 ±9.9 | -0.176 | 0.861 |
| DBP (mmHg) | 63.4 ±7.6 | 63.5 ±8.0 | 0.172 | 0.864 | 64.7 ±8.4 | 64.0 ±7.7 | -0.828 | 0.408 |
| CRF (km/h) | 10.1 ±0.9 | 10.0 ±1.0 | -0.322 | 0.747 | 9.4 ±0.7 | 9.3 ±0.8 | -1.225 | 0.221 |
